# Relationships and Predictive Capabilities of Jump Assessments to Soccer-Specific Field Test Performance in Division I Collegiate Players

**DOI:** 10.3390/sports4040056

**Published:** 2016-12-03

**Authors:** Robert G. Lockie, Alyssa A. Stage, John J. Stokes, Ashley J. Orjalo, DeShaun L. Davis, Dominic V. Giuliano, Matthew R. Moreno, Fabrice G. Risso, Adrina Lazar, Samantha A. Birmingham-Babauta, Tricia M. Tomita

**Affiliations:** 1 Department of Kinesiology, California State University, Fullerton, CA 92831, USA; ashley.orjalo@csu.fullerton.edu; 2 Department of Kinesiology, California State University, Northridge, CA 91330, USA; alyssa.stage.634@my.csun.edu (A.A.S.); john.stokes.91@my.csun.edu (J.J.S.); deshaun.davis.907@my.csun.edu (D.L.D.); dominic.giuliano.871@my.csun.edu (D.V.G.); matthew.moreno.595@my.csun.edu (M.R.M.); fabrice.risso.893@my.csun.edu (F.G.R.); adrina.lazar.957@my.csun.edu (A.L.); samantha.birminghambabauta.162@my.csun.edu (S.A.B.-B.); tricia.tomita.228@my.csun.edu (T.M.T.)

**Keywords:** association football, men’s soccer, leg power, vertical jump, standing broad jump, triple hop, linear speed, repeated-sprint ability, asymmetry

## Abstract

Leg power is an important characteristic for soccer, and jump tests can measure this capacity. Limited research has analyzed relationships between jumping and soccer-specific field test performance in collegiate male players. Nineteen Division I players completed tests of: leg power (vertical jump (VJ), standing broad jump (SBJ), left- and right-leg triple hop (TH)); linear (30 m sprint; 0–5 m, 5–10 m, 0–10, 0–30 m intervals) and change-of-direction (505) speed; soccer-specific fitness (Yo-Yo Intermittent Recovery Test Level 2); and 7 × 30-m sprints to measure repeated-sprint ability (RSA; total time (TT), performance decrement (PD)). Pearson’s correlations (*r*) determined jump and field test relationships; stepwise regression ascertained jump predictors of the tests (*p* < 0.05). All jumps correlated with the 0–5, 0–10, and 0–30 m sprint intervals (*r* = −0.65–−0.90). VJ, SBJ, and left- and right-leg TH correlated with RSA TT (*r* = −0.51–−0.59). Right-leg TH predicted the 0–5 and 0–10 m intervals (R^2^ = 0.55–0.81); the VJ predicted the 0–30 m interval and RSA TT (R^2^ = 0.41–0.84). Between-leg TH asymmetry correlated with and predicted left-leg 505 and RSA PD (*r* = −0.68–0.62; R^2^ = 0.39–0.46). Improvements in jumping ability could contribute to faster speed and RSA performance in collegiate soccer players.

## 1. Introduction

Soccer is a complex, intermittent sport that stresses an athlete’s physiological and technical capacities [1]. There has been much less analysis of the characteristics of male collegiate soccer players from the USA [2,3,4,5], especially when compared to soccer players from different levels of play in Europe. However, upon reviewing this literature [2,3,4,5], it is evident that the physiological capacities required by male collegiate players match those of soccer players from other areas of the world. This includes aerobic and anaerobic fitness, linear and change-of-direction (COD) speed, and strength and power [1,6]. One aspect of performance that is essential for soccer players is leg power. Leg power is often assessed via the use of maximal jump tests [7]. A direct relationship between leg power, jumping ability and soccer performance can be seen in players when they are challenging the ball when it is in the air [8]. However, leg power and jumping ability can also contribute to other aspects of athletic performance important for soccer players.

Numerous studies have investigated relationships between leg power as measured by jump tests and athletic performance in a range of field tests in different populations. This is important as it could be surmised that enhanced performance in a particular jump test could then be related to a change in a certain physiological capacity [9,10]. For example, several investigations have documented significant correlations between better performance in jump tests (e.g., vertical jump (VJ), bilateral and unilateral standing broad jump (SBJ), squat jumps, and drop jumps) and faster linear speed over distances from 5–100 meters (m) [11,12,13,14,15,16,17]. Performance in certain jump tests has also been found to relate to COD speed. For example, Lockie et al. [12] illustrated that a better-performed unilateral SBJ from both the left and right legs was significantly related to faster performance in the 505 COD speed test and modified T-test (correlation coefficient *r* = −0.37–−0.64) in recreational male team sport athletes. Specific to collegiate soccer players, McFarland et al. [14] found that Division II female (*r* = −0.50–−0.76) but not male (*r* = −0.16–−0.32) players demonstrated significant relationships between the VJ and squat jump with pro-agility and T-test times. It could therefore be expected that jump performance should relate to linear speed, and possibly COD speed, in Division I male collegiate soccer players. However, to the author’s knowledge there is no research that has investigated these relationships in this specific population.

In addition to the linear and COD speed, soccer players also require a high aerobic capacity [1], and the ability to complete repeated maximal or near-maximal sprints with incomplete recovery periods [18], which is termed repeated-sprint ability (RSA) [19]. There has been some analysis of the relationship between jump tests and endurance-based running tests and RSA. The VJ (*r* = −0.63) and squat jump (*r* = −0.58) have been found to correlate with the total time (TT) in a RSA test featuring 10 30 m sprints with a 180° direction change run in 30 second (s) cycles in male and female teenage basketball players [20]. In under-18 male soccer players, Spencer et al. [21] found large correlations between a VJ completed with and without arm swing (*r* > 0.70) and TT from an RSA test incorporating 6 × 30 m sprints completed in 30 s cycles. In contrast to this, VJ height did not significantly correlate to the distance run in the Yo-Yo Intermittent Recovery Test (YYIRT) Level 1 (*r* = 0.50) in amateur male soccer players [22], and Rampinini et al. [23] discovered that the VJ did not relate to important measures of soccer match performance in elite European soccer players. The important measures of match performance defined by Rampinini et al. [23] included the total distance run, total high-intensity (runs with a speed above 14 kilometers per hour (km·h^−1^)) and very-high-intensity (running speed above 19.8 km·h^−1^) running distance, and total sprinting (running speed above 25.2 km·h^−1^) distance completed during a match. Thus, the relationships between jump tests and high-intensity running remain unclear, and this is particularly true in Division I collegiate male soccer players.

This issue is further exacerbated when considering how jump tests can be used for physiological monitoring of training load [24,25]. Documenting relationships between jump performance and certain soccer-specific tests could provide an indication of what physiological parameters could be impaired if there is a decline in jumping ability due to neuromuscular fatigue. For example, in an analysis of Australian football players, Mooney et al. [24] stated that neuromuscular fatigue measured by the relationship between flight time and contact time in a VJ negatively affected the degree of high-intensity running completed by players during a match. In elite female soccer players, the decrease in performance for a VJ and 20 m sprint immediately after a soccer match were similar (4% and 3%, respectively), indicating an analogous compromise in the ability to generate force in both of these actions [26]. Collectively considering the results of these studies [20,21,22,23,24,26], it appears imperative to document the potential influence that jump performance has with performance in high-intensity running field tests specific to Division I collegiate soccer players. This will not only provide information as to what aspects of performance could be enhanced with improved leg power shown through jumping, but also those that may be impacted if jump performance is impaired.

Therefore, this study investigated the relationships between jump tests and performance in soccer-specific field tests. Players were assessed in bilateral VJ, bilateral and unilateral SBJ, and left- and right-leg triple-hop (TH). The soccer-specific field tests include a 30 m sprint (0–5 m, 0–10 m, and 0–30 m intervals), 505 from each leg, COD deficit derived from the 505 [27], YYIRT Level 2 (YYIRT2), and an RSA test (7 × 30 m sprints in 20 s cycles) [4]. It was hypothesized that the jump tests would correlate with and predict performance in all the field tests. However, the relationships would be stronger in those tests that required a higher speed running (i.e., the linear and COD speed tests, RSA test).

## 2. Materials and Methods

### 2.1. Subjects

Nineteen players (age: 20.53 ± 1.50 years; height = 1.81 ± 0.06 m; body mass = 77.57 ± 6.14 kg) from the same Division I collegiate men’s soccer team were recruited for this study. The subjects were required to be: a member of the playing squad; over 18 years of age; and injury-free and currently completing full training at the time of testing. This sample included two goalkeepers; six defenders; five midfielders; and six forwards. The data used in this study arose as a condition of player monitoring in which player activities were measured during the pre-season [4,6,28]. Thus, the institutional ethics committee approved the use and analysis of pre-existing data. The study still conformed to the recommendations of the Declaration of Helsinki, and all subjects received a clear explanation of the study procedures. Each player had also completed the university-mandated physical examination, and read and signed the university consent and medical forms for participation in collegiate athletics.

### 2.2. Procedures

The procedures used in this study were documented in detail by Lockie et al. [4], and the reader is directed to this manuscript to review the specifics of the test design, equipment, and administration. To briefly surmise, testing was conducted within the team’s pre-season period. The jump tests were conducted within one gym session. The running assessments were completed prior to two field training sessions separated by 48 h. Session 1 incorporated the 30 m sprint, 505 COD speed test, and RSA test; session 2 involved the YYIRT2. The VJ, SBJ, and TH were all measured in meter, and provided indirect assessments of bilateral and unilateral leg power [4,6,12,14,29,30]. Additional measures for this study included the calculation of the asymmetry measured as a percentage between the legs in the SBJ and TH, which was derived via the equation: (better performing leg − lesser performing leg)/better performing leg × 100 [12]. The 30 m sprint (measured in second) provided a measure of linear acceleration (0–5 m and 0–10 m intervals) and maximal velocity (0–30 m interval) specific to soccer [4,6]. The 505 assessed COD speed, by measuring the time taken for the subject to execute a 180° cut when either the left or right leg was used as the turning leg [4,27,31]. An additional 505 variable that was included in this study was the COD deficit for each leg, which was calculated via the formula: 505 time − 0–10 m time [27], which provided another measure of COD ability. The 0–10 m time was taken from the 30 m sprint. All variables from the 505 were measured in second. The YYIRT2 assessed aerobic and anaerobic endurance specific to soccer, with the maximum level attained providing a distance measure in m [4,32]. The RSA test used in this study was designed by Lockie et al. [4]. The two variables drawn from this test were TT for the seven sprints measured in second, and performance decrement (PD; measured as a percentage) from the first to last sprint, calculated via the equation: percent change (%) = ([last sprint time − first sprint time]/first sprint time) × 100.

### 2.3. Statistical Analysis

All statistical analyses were computed using the Statistics Package for Social Sciences (Version 22.0; IBM Corporation, New York, NY, USA). Descriptive statistics (mean ± standard deviation (SD); 95% confidence intervals (CI)) were calculated for each test parameter, and stem-and-leaf plots confirmed a normal distribution in data for each variable. Pearson’s two-tailed correlation analysis determined relationships between the jump tests (VJ, SBJ, and TH) with the athletic performance tests (30-m sprint, 505, YYIRT, and RSA test). An alpha level of *p* < 0.05 was required for significance. The correlation strength was designated as: an *r* between 0 to 0.3, or 0 to −0.3, was considered small; 0.31 to 0.49, or −0.31 to −0.49, moderate; 0.5 to 0.69, or −0.5 to −0.69, large; 0.7 to 0.89, or −0.7 to −0.89, very large; and 0.9 to 1, or −0.9 to −1, near perfect for relationship prediction [33]. Stepwise multiple regression analyses (*p* < 0.05) were conducted for the soccer-specific field tests (each acted as a dependent variable) to illustrate whether a particular jump test predicted test performance.

## 3. Results

Table 1 shows the descriptive data for each of the performance tests. The correlation data for the VJ and TH is displayed in Table 2, while the SBJ data is shown in Table 3. All significant correlations for the jump performance tests were negative, which indicated a faster time in the particular sprint test related to a superior jump performance. The 0–5 m sprint time had large relationships with the VJ, all SBJ tests, and the left-leg TH, and a very large relationship with the right-leg TH. The 0–10 m sprint time exhibited large relationships with left- and right-leg SBJ, very large relationships with VJ, SBJ, and left-leg TH, and a near-perfect relationship with right-leg TH. Large relationships were found between the 0–30 m sprint time and the left- and right-leg SBJ; very large relationships were documented with VJ, SBJ and left- and right-leg TH. RSA TT had significant, large correlations with VJ, SBJ, and left- and right-leg TH. There were no significant relationships between any jump test and the 505 or COD deficit from either leg, the YYIRT2, or RSA PD.

With regards to the asymmetry measures for the TH (Table 2) and unilateral SBJ (Table 3), only the TH asymmetry displayed any significant relationships. There were positive, large correlations with the left-leg 505 and COD deficit, indicating that a larger asymmetry was related to greater (i.e., slower) speed test times. There was also a negative, large correlation with RSA PD, which suggested that a larger asymmetry was related to a smaller PD in the RSA test.

The stepwise regression data is shown in Table 4. The 0–5 m and 0–10 m sprint intervals were both predicted by the right-leg TH, with 55% and 81% explained common variance, respectively. The 0–30 m sprint interval and RSA TT were predicted by the VJ, with 84% and 41% explained variance, respectively. The TH asymmetry predicted the left-leg 505 (46% explained variance) and COD deficit (42% explained variance), as well as the RSA PD (39% explained variance).

## 4. Discussion

This study investigated the relationships between leg power as measured via jump assessments (VJ, SBJ, and TH) and performance in soccer-specific field tests (30 m sprint, 505 COD speed test, YYIRT2, and RSA test). In line with the study’s hypothesis, there were significant correlations and predictive relationships between the VJ, SBJ, and TH, with a linear speed over 30 m, as well as the TT from the RSA test. The between-leg asymmetry measured from the TH was also related to and predicted the left-leg 505 and COD deficit and RSA PD. However, counter to the study’s hypothesis, there were no significant relationships between the jump tests and right-leg 505 and COD deficit, and high-intensity running as measured by the YYIRT2. As will be discussed, these results have several implications for the strength and conditioning coaches of Division I collegiate male soccer players.

As previously acknowledged, numerous studies have detailed significant relationships between jumping ability and linear sprint speed over a variety of distances [11,12,13,14,15,16,17]. The results from this study provided support for these studies, as each jump test significantly correlated with all intervals from the 30 m sprint (*r* = −0.54–−0.90). These relationships occur as both jumping and sprinting place a great emphasis on the stretch-shortening and power production capacities of the leg muscles [15,34], in both the horizontal [35,36] and vertical [15,37] planes. Maximal velocity sprinting in particular places a relatively greater emphasis on vertical force production [37] and the stretch-shortening cycle capacities of the leg muscles [15,34], which helps illustrate why the VJ predicted the 0–30 m sprint performance (R^2^ = 0.84). Enhancements in leg power and jumping ability through specific training should contribute to the faster sprint speed in Division I collegiate male soccer players. Indeed, previous research has verified that improvements in leg power following specific speed training can lead to faster sprint acceleration and maximal velocities in trained individuals [9,10,30,38]. In addition to this, any decrement in jumping ability due to neuromuscular fatigue could negatively impact sprint performance.

Interestingly, the strongest correlations with the sprint intervals occurred for the right-leg TH (0–5 m *r* = −0.74; 0–10 m *r* = −0.91; 0–30 m *r* = −0.88). The right-leg TH also predicted the 0–5 m (R^2^ = 0.55) and 0–10 m (R^2^ = 0.81) sprint performances. The TH is not as extensively used as other jump tests such as the VJ and SBJ in performance-based research [4,30], but has been featured in clinical research as a rehabilitation assessment [39,40]. Hamilton et al. [29] described the TH test as a valid predictor for leg power, which has the added benefit of providing a unilateral measure of jump performance. Given the cyclic, unilateral nature of sprinting [37], it is notable that significant relationships were shown with both sprint acceleration (0–5 m and 0–10 m intervals) and maximum velocity (0–30 m interval). These results suggest that coaches could consider utilizing the TH as a leg power assessment in collegiate soccer players.

The potential value of using the TH as a leg power assessment could also be seen with the between-leg asymmetries measured from this test. A greater between-leg TH asymmetry was related to slower left-leg 505 and COD deficit performance. The 505 and COD deficit isolate the ability to change direction from each leg [27], and differences in the ability to cut from either leg in a test such as the 505 can negatively impact the COD speed [41]. Potentially, the TH, which, as stated, has been traditionally used a functional lower-body test during rehabilitation [29,39,40], could also provide some indication of COD speed. However, the TH asymmetry did not relate to the linear speed as measured by the 30 m sprint, or the right-leg 505 and COD deficit, and the unilateral SBJ asymmetry did not relate to the 30 m sprint, 505 or COD deficit measured from either leg. Lockie et al. [12] also found that asymmetries measured by the unilateral VJ, SBJ, and lateral jump did not relate to multidirectional speed as measured by a 20 m sprint, 505, and modified T-test. Interestingly, for both the SBJ (4.69% ± 4.14%) and TH (4.36% ± 3.52%), the players from this team were well below the suggested clinically significant asymmetry level of 15% [42]. This suggests that any asymmetries as measured by the unilateral SBJ and TH for the subjects in this study would generally not impact unilateral activities such as sprinting and changing direction.

There were also no significant relationships between VJ height, and SBJ and TH distance, and the 505 or COD deficit in the current study. McFarland et al. [14] also found that the VJ did not relate to the COD speed measured by the pro-agility (*r* = −0.30) and T-test (*r* = −0.16) in Division II male soccer players. Many physiological capacities contribute to COD speed, including leg power and strength, bilateral asymmetries, and the technique associated with the COD [43]. Furthermore, leg power is directionally specific [44], so potentially a jump that features lateral projection may better relate to the 505 performance in soccer players. Delaney et al. [45] found that dominant-leg lateral jump performance was a predictor of the 505 performed from the non-dominant leg (R^2^ = 0.40) in professional rugby league players. Lockie et al. [12] detailed that left- and right-leg lateral jump performance was related to 505 performance in recreational male team sport athletes (*r* = −0.47–−0.59), while the left-leg lateral jump predicted both the left- (R^2^ = 0.22) and right-leg (R^2^ = 0.34) 505. Future research should investigate lateral jump performance in Division I collegiate male soccer players and whether it provides predictive capabilities as it relates to COD speed. With regards to the COD deficit, Nimphius et al. [27] stated that this may provide an alternate measure of COD ability by removing the influence that linear speed may have on a COD speed test such as the 505. This may provide some reason as to why there were no significant relationships with the COD deficit, as the influence of stretch-shortening capacities prevalent in the jump tests may not be present in the COD deficit. The results from this study showed that jump height measured by the VJ, or distance as a measure provided by the SBJ and TH, does not relate to or predict COD speed as measured by the 505 in Division I collegiate male soccer players. However, between-leg TH asymmetry may provide some useful information about lower-body function that could influence COD speed.

Maximal sprint velocity is an important contributor to the TT measured in an RSA test [18]. Given the influence of leg power on maximal sprinting [11,12,13,14,15], it is not surprising that there were significant correlations between selected jump tests (VJ, SBJ, left- and right-leg TH) and RSA TT. The VJ also predicted RSA TT (R^2^ = 0.41), although the explained variance between the VJ and RSA TT (41%) was less than that for the 30 m sprint (84%). The physiological demands of an RSA test exceed that of a single maximal sprint [19], which highlights why there was a difference in the explained variance. Potentially, improvements in bilateral (VJ and SBJ) and unilateral (TH) jumping could contribute to an improvement in RSA, especially if the maximal sprint velocity is increased.

RSA PD did not correlate with any jump performance test, but did correlate with and was predicted by the between-leg TH asymmetry. A greater TH asymmetry was related to a higher PD in the RSA test. A higher PD provides a measure of fatigue in soccer players during an RSA test [46]. Fatigue can exacerbate between-leg asymmetries in a VJ [47] and during an incremental running test to exhaustion [48] in healthy individuals, which provides some indication as to why TH asymmetry was related to RSA PD. Nonetheless, several factors contribute to the decrease in sprint performance associated with an RSA test, including oxidative capacity, phosphocreatine recovery, hydrogen buffering, and muscle activation and recruitment strategies [19,49]. In isolation, jump performance as a measure of leg power does not indicate RSA PD, as jumping provides a different measure of physiological capacity in a soccer player. However, greater between-leg asymmetries in power as measured by the TH may be related to decreased sprint performance with the onset of fatigue.

The YYIRT2 heavily taxes both the aerobic and anaerobic capacities of an individual [32]. Some of the physiological responses to the YYIRT2 include elevated heart rate and oxygen uptake, and blood lactate, hydrogen ion, and bicarbonate accumulation [50]. Given the range of physiological responses detailed by Rampinini et al. [50], there would be a number of limiting factors to YYIRT2 exceeding the influence of leg power as measured by jump tests, or the between-leg SBJ and TH asymmetries. The findings from this study provide support to previous research that identified limited relationships between VJ height and high-intensity running performance in soccer players [22,23]. However, rather than considering VJ height as the measure of performance when investigating relationships to high-intensity running, it may be more valuable for strength and conditioning coaches and sport scientists to instead utilize the ratio between flight and contact time as the measure of neuromuscular performance. This is the VJ measure commonly used to monitor neuromuscular fatigue, which can manifest in a reduced ability to produce high-speed running actions during match play in team sport athletes [24,25]. Future research should further investigate relationships between VJ flight and contact time ratios and high-intensity running tests in Division I collegiate male soccer players.

There are certain limitations with this research that should be discussed. Only one Division I collegiate men’s soccer squad was analyzed in this study. This limits the generalizability of the current results, and indeed the data may reflect the training practices of this team. Ideally, future research could integrate multiple collegiate squads in the analysis of relationships between jump assessments and soccer-specific field test performance. The lateral jump was not analyzed in this study, and previous research suggests that lateral jump performance may have relationships with linear and COD speed [12,45]. As noted, VJ flight–to–contact time ratios were also not considered in this study. These are both jump assessments that could be quantified in forthcoming collegiate soccer research. Furthermore, only one assessment of the COD speed (i.e., the 505) and RSA (i.e., 7 × 30 m sprints completed in 20 s cycles) test was measured in this research. Future studies could include other measures of COD speed and RSA to elucidate whether any relationships with jump performance are assessment-specific. Nonetheless, within the context of these limitations, this study demonstrated that 0–5 m, 0–10 m, and 0–30 m sprint performances, and TT from a soccer-specific RSA test, were related to jump tests such as the VJ, bilateral and unilateral SBJ, and light- and right-leg TH. Further, between-leg TH asymmetry was related to left-leg 505 and COD deficit and RSA PD.

## 5. Conclusions

The results from this study showed that there were select relationships between the jump tests and performance in the soccer-specific field tests. The VJ, bilateral and unilateral SBJ, and TH were related to all the measured intervals (0–5 m, 0–10 m, and 0–30 m) in the 30 m sprint, while the VJ, bilateral SBJ, and left- and right-leg TH correlated with RSA TT. The 0–5 m and 0–10 m sprint intervals were predicted by the right-leg TH; the 0–30 sprint interval and RSA TT were predicted by the VJ. A greater between-leg asymmetry as measured by the TH related to and predicted left-leg 505 and COD deficit and RSA PD. Improvements in these jumps through specific training could engender crossover into linear speed and RSA performance. It could also be surmised that any decrements in VJ, SBJ, and TH indicative of neuromuscular fatigue could negatively affect sprinting ability. Reducing between-leg differences in the TH may have some positive influence on the COD ability and RSA performance. Lastly, strength and conditioning coaches should also consider using the TH as an assessment for leg power, given the significant relationships found with linear speed over 30 m, COD speed as measured by the 505, and TT and PD from the RSA test.

## Figures and Tables

**Table 1:** Physiological characteristics (mean ± standard deviation (SD); 95% confidence intervals (CI)) of Division I collegiate men’s soccer players as measured by: vertical jump; bilateral and unilateral standing broad jump; left- and right-leg triple hop; asymmetries between the legs for the standing broad jump and triple hop; 30 m sprint (0–5 m, 0–10 m, and 0–30 m intervals); 505 change-of-direction speed test from the left and right legs, and the COD deficit for each leg; the Yo-Yo Intermittent Recovery Test Level 2; and the total time and performance decrement from the first to last sprint from a repeated sprint ability (RSA) test.

Field Test	Mean ± SD (95% CI)
Vertical Jump (m)	0.65 ± 0.08 (0.62–0.69)
Standing Broad Jump (m)	2.41 ± 0.22 (2.30–2.52)
Left-Leg Standing Broad Jump (m)	2.16 ± 0.19 (2.07–2.25)
Right-Leg Standing Broad Jump (m)	2.10 ± 0.20 (2.00–2.19)
Unilateral Standing Broad Jump Asymmetry (%)	4.69 ± 4.14 (2.70–6.69)
Left-Leg Triple Hop (m)	6.89 ± 0.58 (6.58–7.19)
Right-Leg Triple Hop (m)	6.98 ± 0.72 (6.59-7.36)
Triple Hop Asymmetry (%)	4.36 ± 3.52 (2.48–6.24)
0–5 m Interval (s)	1.006 ± 0.052 (0.981–1.031)
0–10 m Interval (s)	1.719 ± 0.063 (1.689–1.749)
0–30 m Interval (s)	4.110 ± 0.150 (4.038–4.182)
Left-Leg 505 (s)	2.237 ± 0.143 (2.166–2.309)
Left-Leg COD Deficit (s)	0.549 ± 0.144 (0.478–0.621)
Right-Leg 505 (s)	2.203 ± 0.088 (2.159–2.246)
Right-Leg COD Deficit (s)	0.512 ± 0.104 (0.460–0.563)
Yo-Yo Intermittent Recovery Test Level 2 (m)	1048.78 ± 365.91 (865.62–1229.74)
RSA Total Time (s)	31.937 ± 1.031 (31.424–32.450)
RSA Performance Decrement (%)	5.98 ± 4.45 (3.76–8.19)

**Table 2:** Correlations (*r* and 95% confidence intervals (CI)) between the vertical jump (VJ), left- and right-leg triple-hop (TH), and asymmetry between the legs in the TH, with 0–5 m, 0–10 m, and 0–30 m sprint performance, left- and right-leg 505 and change-of-direction (COD) deficit, Yo-Yo Intermittent Recovery Test Level 2 (YYIRT2) distance, and repeated-sprint ability (RSA) total time (TT) and performance decrement (PD) in Division I collegiate men’s soccer players. R^2^ = explained variance; *p* = significance.

Variable	Statistics	VJ	Left-Leg TH	Right-Leg TH	TH Asymmetry
0–5 m	*r*	−0.55 *	−0.62 *	−0.74 *	−0.27
	95% CI	−0.80–−0.13	−0.84–−0.23	−0.89–−0.43	−0.64–0.21
	R^2^	0.31	0.38	0.55	0.07
	*p*	0.01	0.01	<0.01	0.32
0–10 m	*r*	−0.74 *	−0.78 *	−0.90 *	−0.23
	95% CI	−0.89–−0.43	−0.91–−0.50	−0.96–−0.75	−0.62–0.25
	R^2^	0.55	0.61	0.81	0.05
	*p*	<0.01	<0.01	<0.01	0.40
0–30 m	*r*	−0.77 *	−0.75 *	−0.88 *	−0.10
	95% CI	−0.91–−0.48	−0.90–−0.45	−0.95–−0.71	−0.53–0.37
	R^2^	0.59	0.56	0.77	0.01
	*p*	<0.01	<0.01	<0.01	0.72
Left-Leg 505	*r*	−0.04	−0.37	−0.03	0.68 *
	95% CI	−0.49–0.42	−0.71–0.10	−0.48–0.43	0.32–0.86
	R^2^	<0.01	0.14	<0.01	0.46
	*p*	0.88	0.17	0.92	<0.01
Left-Leg COD Deficit	*r*	0.19	−0.10	0.25	0.65 *
	95% CI	−0.29–0.59	−0.53–0.37	−0.23–0.63	0.27–0.85
	R^2^	0.04	0.01	0.06	0.42
	*p*	0.46	0.71	0.37	<0.01
Right-Leg 505	*r*	0.03	−0.21	−0.03	0.30
	95% CI	−0.43–0.48	−0.61–0.27	−0.48–0.43	−0.18–0.66
	R^2^	<0.01	0.04	<0.01	0.09
	*p*	0.91	0.45	0.92	0.27
Right-Leg COD Deficit	*r*	0.31	0.03	0.32	0.43
	95% CI	−0.17–0.67	−0.43–0.48	−0.16–0.68	−0.04–0.74
	R^2^	0.10	<0.01	0.10	0.18
	*p*	0.21	0.92	0.24	0.11
YYIRT2	*r*	−0.06	0.07	0.04	−0.20
	95% CI	−0.50–0.41	−0.40–0.57	−0.42–0.49	−0.60–0.28
	R^2^	<0.01	0.01	<0.01	0.04
	*p*	0.83	0.81	0.90	0.48
RSA TT	*r*	−0.55 *	−0.57 *	−0.59 *	0.05
	95% CI	−0.80–−0.13	−0.81–−0.16	−0.82–−0.18	−0.41–0.50
	R^2^	0.31	0.32	0.35	<0.01
	*p*	0.02	0.03	0.02	0.85
RSA PD	*r*	0.15	0.23	0.04	−0.62 *
	95% CI	−0.33–0.57	−0.25–0.62	−0.42–0.49	−0.41–0.50
	R^2^	0.02	0.05	<0.01	0.38
	*p*	0.55	0.41	0.90	0.01

* Significant (*p* < 0.05) relationship between the two variables.

**Table 3:** Correlations (*r* and 95% confidence intervals (CI)) between the bilateral standing broad jump (SBJ), left- and right-leg SBJ, and asymmetry between the legs in the unilateral SBJ, with 0–5 m, 0–10 m, and 0–30 m sprint performance, left- and right-leg 505 and change-of-direction (COD) Deficit, Yo-Yo Intermittent Recovery Test Level 2 (YYIRT2) distance, and repeated-sprint ability (RSA) total time (TT) and performance decrement (PD) in Division I collegiate men’s soccer players. R^2^ = explained variance; *p* = significance.

Variable	Statistics	SBJ	Left-Leg SBJ	Right-Leg SBJ	SBJ Asymmetry
0–5 m	*r*	−0.55 *	−0.56 *	−0.55 *	0.05
	95% CI	−0.80–−0.13	−0.81–−0.14	−0.80–−0.13	−0.07–0.78
	R^2^	0.31	0.31	0.31	<0.01
	*p*	0.01	0.01	0.02	0.84
0–10 m	*r*	−0.71 *	−0.66 *	−0.67 *	0.14
	95% CI	−0.88–−0.38	−0.86–−0.29	−0.86–−0.31	−0.33–0.56
	R^2^	0.50	0.44	0.45	0.02
	*p*	<0.01	<0.01	<0.01	0.56
0–30 m	*r*	−0.70 *	−0.54 *	−0.68 *	0.32
	95% CI	−0.88–−0.36	−0.80–−0.11	−0.87–−0.33	−0.16–0.67
	R^2^	0.49	0.29	0.46	0.10
	*p*	<0.01	0.02	<0.01	0.19
Left-Leg 505	*r*	−0.11	0.18	−0.02	0.45
	95% CI	−0.54–0.36	−0.30–0.59	−0.47–0.44	−0.01–0.75
	R^2^	0.01	0.03	<0.01	0.20
	*p*	0.65	0.48	0.93	0.06
Left-Leg COD Deficit	*r*	0.13	0.31	0.16	0.29
	95% CI	−0.34–0.55	−0.17–0.67	−0.32–0.57	−0.19–0.65
	R^2^	0.02	0.10	0.03	0.08
	*p*	0.62	0.21	0.53	0.25
Right-Leg 505	*r*	−0.04	0.12	−0.12	0.38
	95% CI	−0.49–0.42	−0.35–0.54	−0.54–0.35	−0.09–0.71
	R^2^	<0.01	0.01	0.01	0.14
	*p*	0.88	0.64	0.65	0.12
Right-Leg COD Deficit	*r*	0.14	0.33	0.14	0.26
	95% CI	−0.34–0.56	−0.15–0.68	−0.34–0.56	−0.22–0.64
	R^2^	0.02	0.11	0.02	0.07
	*p*	0.59	0.19	0.58	0.30
YYIRT2	*r*	−0.09	−0.11	0.03	−0.22
	95% CI	−0.52–0.38	−0.54–0.36	−0.43–0.48	−0.61–0.26
	R^2^	0.01	0.01	<0.01	0.05
	*p*	0.72	0.65	0.89	0.39
RSA TT	*r*	−0.51 *	−0.28	−0.46	0.36
	95% CI	−0.78–−0.07	−0.62–0.20	−0.76–−0.01	−0.11–0.70
	R^2^	0.26	0.08	0.21	0.13
	*p*	0.03	0.26	0.05	0.14
RSA PD	*r*	0.09	0.17	0.29	−0.03
	95% CI	−0.38–0.52	−0.31–0.58	−0.19–0.66	−0.48–0.43
	R^2^	0.01	0.03	0.08	<0.01
	*p*	0.73	0.49	0.25	0.91

* Significant (*p* < 0.05) relationship between the two variables.

**Table 4:** Stepwise linear regression analysis between 0–5 m, 0–10 m, and 0–30 m sprint intervals, and repeated-sprint ability (RSA) total time (TT) and performance decrement (PD) (dependent variables), and vertical jump (VJ), standing broad jump (SBJ), left- and right-leg SBJ, left- and right-leg triple-hop (TH), and SBJ and TH between-leg asymmetry.

Variables	*r*	R^2^	Significance
0–5 m sprint interval			
Right-leg TH	0.74	0.55	*p* < 0.01
0–10 m sprint interval			
Right-leg TH	0.90	0.81	*p* < 0.01
0–30 m sprint interval			
VJ	0.92	0.84	*p* < 0.01
Left-leg 505			
TH Asymmetry	0.68	0.46	*p* = 0.01
Left-leg COD Deficit			
TH Asymmetry	0.65	0.42	*p* = 0.01
RSA TT			
VJ	0.64	0.41	*p* = 0.01
RSA PD			
TH Asymmetry	0.62	0.39	*p* = 0.01

## Abbreviations

The following abbreviations are used in this manuscript:

COD: Change-of-direction
VJ: Vertical jump
SBJ: Standing broad jump
m: Meter
*r*: Correlation coefficient
RSA: Repeated-sprint ability
TT: Sum of all sprint times from an RSA test
s: Second
YYIRT: Yo-Yo Intermittent Recovery Test
km·h^−1^: Kilometers per hour
TH: Triple hop
YYIRT2: Yo-Yo Intermittent Recovery Test Level 2
PD: Sprint time decrement measured as percentage from first to last sprint in RSA test
SD: Standard deviation
CI: Confidence intervals
*p*: Significance
